# Subthalamic nucleus-deep brain stimulation improves autonomic dysfunctions in Parkinson’s disease

**DOI:** 10.1186/s12883-022-02651-z

**Published:** 2022-03-31

**Authors:** Feng Zhang, Feng Wang, Cong-Hui Li, Ji-Wei Wang, Chun-Lei Han, Shi-Ying Fan, Dong-Mei Gao, Yu-Jing Xing, Chen Yang, Jian-Guo Zhang, Fan-Gang Meng

**Affiliations:** 1 Department of Neurosurgery, the First Hospital of Hebei Medical University, Shijiazhuang, 050031 Hebei China; 2 Departments of Neurosurgery, The First Affiliated Hospital, Zhejiang University School of Medicine, Hangzhou, 310000 Zhejiang China; 3 Departments of Neurosurgery, General Hospital of Ningxia Medical University, Yinchuan, 750004 Ningxia China; 4 Department of Neurosurgery, Beijing Tiantan Hospital, Capital Medical University, Beijing, 100070 China; 5 Beijing Neurosurgical Institute, Capital Medical University, Beijing, 100070 China; 6 Beijing Key Laboratory of Neurostimulation, Beijing, 100070 China; 7 Chinese Institute for Brain Research, Beijing, 102206 China

**Keywords:** Parkinson’s disease, Deep brain stimulation, Subthalamic nucleus, Autonomic dysfunction, Non-motor symptoms

## Abstract

**Background:**

To study the effects of subthalamic nucleus-deep brain stimulation (STN-DBS) on autonomic dysfunctions in Parkinson’s disease (PD) patients.

**Methods:**

A total of 57 PD patients who underwent bilateral STN-DBS from March to December 2018, were retrospectively analyzed. Preplanned assessments at baseline and postoperatively at 1, 3, and 6 months also included the Scales for Outcomes in Parkinson’s Disease-Autonomic questionnaire (SCOPA-Aut), the Unified Parkinson’s Disease Rating Scale (UPDRS) III score, levodopa equivalent day dose (LEDD), Parkinson’s Disease Quality of Life Scale (PDQ-39), the Hamilton Anxiety Rating Scale (HAMA), and the Hamilton Depression Rating Scale (HAMD).

**Results:**

The SCOPA-Aut scores improved significantly [14.59% (18.32%), 24.00% (27.05%), 22.16% (27.07%), all *P* < 0.001] at 1 month, 3 months, and 6 months of STN-DBS, respectively. Analysis of the SCOPA-Aut sub-items showed significant improvements only in urine and thermoregulation sub-items at 6 months after surgery (*P* < 0.001). There was no significant correlation between improvements of SCOPA-Aut scores and improvements of PDQ-39 scores (*P* > 0.05) at 6 months after surgery. SCOPA-Aut scores were positively correlated with age (*r* = 0.428, *P* = 0.001); the improvements of SCCOPA-Aut scores were positively correlated with improvements of HAMA and HAMD scores (HAMA: *r* = 0.325, *P* = 0.015; HAMD: *r* = 0.265, *P* = 0.049) at 6 months after surgery.

**Conclusion:**

STN-DBS improved autonomic dysfunction symptoms of PD patients, and urinary and thermoregulatory sub-items of autonomic dysfunction were improved in the short-term after surgery. There was a close relationship between improved autonomic symptoms and improved anxiety and depression 6 months after surgery. We should therefore direct more attention to autonomic dysfunctions in PD involving detailed preoperative evaluations and postoperative follow-ups, to improve the quality of life of patients.

## Background

Parkinson’s Disease (PD) is a common neurodegenerative disease of the central nervous system. Deep brain stimulation (DBS) is an effective treatment in advanced PD patients [1]. Subthalamic nucleus (STN) DBS has been shown to improve motor symptoms and the quality of life (QOL) [2–4], whereas the effects on nonmotor symptoms (NMS) have been less reported. NMS may have a greater impact on QOL than motor symptoms. Dysfunctions of the autonomic nervous system (gastrointestinal symptoms, urinary symptoms, cardiovascular symptoms, thermoregulation, pupillomotor functions, and sexual functions) are common in PD, and autonomic dysfunctions may appear earlier than major motor symptoms of PD, and significantly impair the QOL. Autonomic dysfunctions are associated with the accumulation of Lewy bodies in the nervous system, and the peripheral autonomic nervous system may be a key route for α-synuclein pathology propagation from the periphery to the central nervous system. PD progression and dopaminergic drug therapy may also aggravate autonomic dysfunctions. The effects of STN-DBS on autonomic symptoms such as sweating, urgency, increased frequency or incontinence, have not been well studied. The loss of central dopamine leads to motor symptoms, which can also lead to autonomic dysfunctions. Thus, we speculated that STN-DBS may improve autonomic dysfunctions. In the present study, we evaluated the effects of bilateral STN-DBS on autonomic dysfunctions during 6 months of chronic stimulation, and determined their relationships with motor symptoms, anxiety, depression, and the QOL.

## Materials and methods

Between March and December 2018, a total of 57 patients were hospitalized at the Department of Neurosurgery of the Beijing Tiantan Hospital Affiliated to Capital Medical University, the First Hospital of Hebei Medical University, and the General Hospital of Ningxia Medical University to optimize a previously-performed STN-DBS, as previously reported [5].

The study was approved by the Medical Ethics Committee of the First Hospital of Hebei Medical University and the Medical Ethics Committee of Beijing Tiantan Hospital Affiliated to Capital Medical University. The ethical principles involved in this research were in accordance with the Declaration of Helsinki, and all patients provided written informed consent, as mentioned in our previous report [5].

### Patient selection

Evaluations were conducted by neurologists specializing in movement disorders. Patients with advanced idiopathic PD were diagnosed based on the diagnostic criteria for PD in China (2016 edition), and PD surgical treatment evaluation criteria [6, 7]. None of the patients had serious cognitive impairments or mental illness. All patients underwent preoperative testing and were analyzed by the levodopa challenge test, confirming that the levodopa response needed to be improved by at least 30%. Those individuals who had complete imaging and scoring data were followed-up on a regular basis. Morphological magnetic resonance imaging was performed to exclude patients with severe cerebral atrophy and ischemic disease.

### Clinical evaluation

Demographic characteristics (age, sex, age at onset, and duration of the disease) and disease severity were assessed by the Unified Parkinson’s Disease Rating Scale (UPDRS)-III scores (range: 0 − 132). The Hoehn-Yahr scale (0 − 5) was used for disease staging. The therapeutic medical regimen was recorded, which calculated the levodopa equivalent dose (LEDD) according to Tomlinson et al. [8]. As part of the preplanned investigations performed at baseline, and after 1, 3, and 6 months of STN-DBS, autonomic symptoms were assessed with the Scopa-Aut questionnaire (0 − 69) [9], consisting of 26 items. It included gastrointestinal symptoms (seven items), urinary symptoms (six items), cardiovascular symptoms (three items), thermoregulation (four items), pupillomotor function (one item), sexual function (two separate items for each sex) [9]. Each item was scored from 0 (never) to 3 (often), except for question 26, which was a yes/no question, and consequently not included in our statistical analysis. SCOPA-Aut scores ranged from 0 to 69, with higher scores expressing more severe symptoms. Anxiety and depression outcomes were assessed using the Hamilton Anxiety Rating Scale (HAMA) (14 parts). The HAMA ranged from 0 to 56 and the Hamilton Depression Rating Scale (HAMD) (24 parts) ranged from 0 to 68. The quality of life (QOL) was assessed using the 39-item Parkinson’s Disease Questionnaire (PDQ-39), ranging from 0 to 124. Postoperative improvement (%) was calculated as the preoperative score—postoperative score)/preoperative score × 100%. Clinical assessments were performed at preoperative baseline (Med-OFF and Med-ON), 1 month after surgery (follow-up 1), 3 months after surgery (follow-up 2), and 6 months after surgery (follow-up 3).

### Surgical procedures

Surgical procedures were conducted as previously described [5].

### Stimulation programming

One month after surgery, we turned on the stimulator and programed the IPG [10], tested the contacts on each electrode, and selected the best stimulation target when the patient obtained satisfactory improvement with minimal side effects. Then, if necessary, the parameters were adjusted using the remote program control. We first used the unipolar stimulation mode, with the following stimulation parameters: the voltage was 1.5 ~ 2.0 V, the frequency was 130 Hz, and the pulse width was 60 ms. We then gradually adjusted the stimulation parameters until the best therapeutic effect was achieved.

### Statistical analyses

All statistical analyses were performed using SPSS statistical software for Windows, version 25.0 (SPSS, Chicago, IL, USA). Continuous variables that followed, or approximately followed, a normal distribution are presented as the mean ± standard deviation ($\overline{x }$± s). Continuous variables that did not follow a normal distribution are presented as the median (M) and interquartile range (IQR). The Friedman test was used for continuous variables that did not follow a normal distribution and the Kruskal–Wallis rank sum test was used for comparisons between multiple groups. The correlation analysis method was used to identify factors influencing the improvement of autonomic dysfunctions after DBS. The statistical significance threshold was fixed at *P* < 0.05.

## Results

### Patient population

As previously reported [5], the study group was comprised of 34 males and 23 females. The LEDD of the 57 preoperative patients was (866.3 ± 357.0) (125–1625) mg/d, and the preoperative Hoehn-Yahr stage was (2.9 ± 0.3) (2 − 4).

### Clinical outcomes

**Fig. 1:**
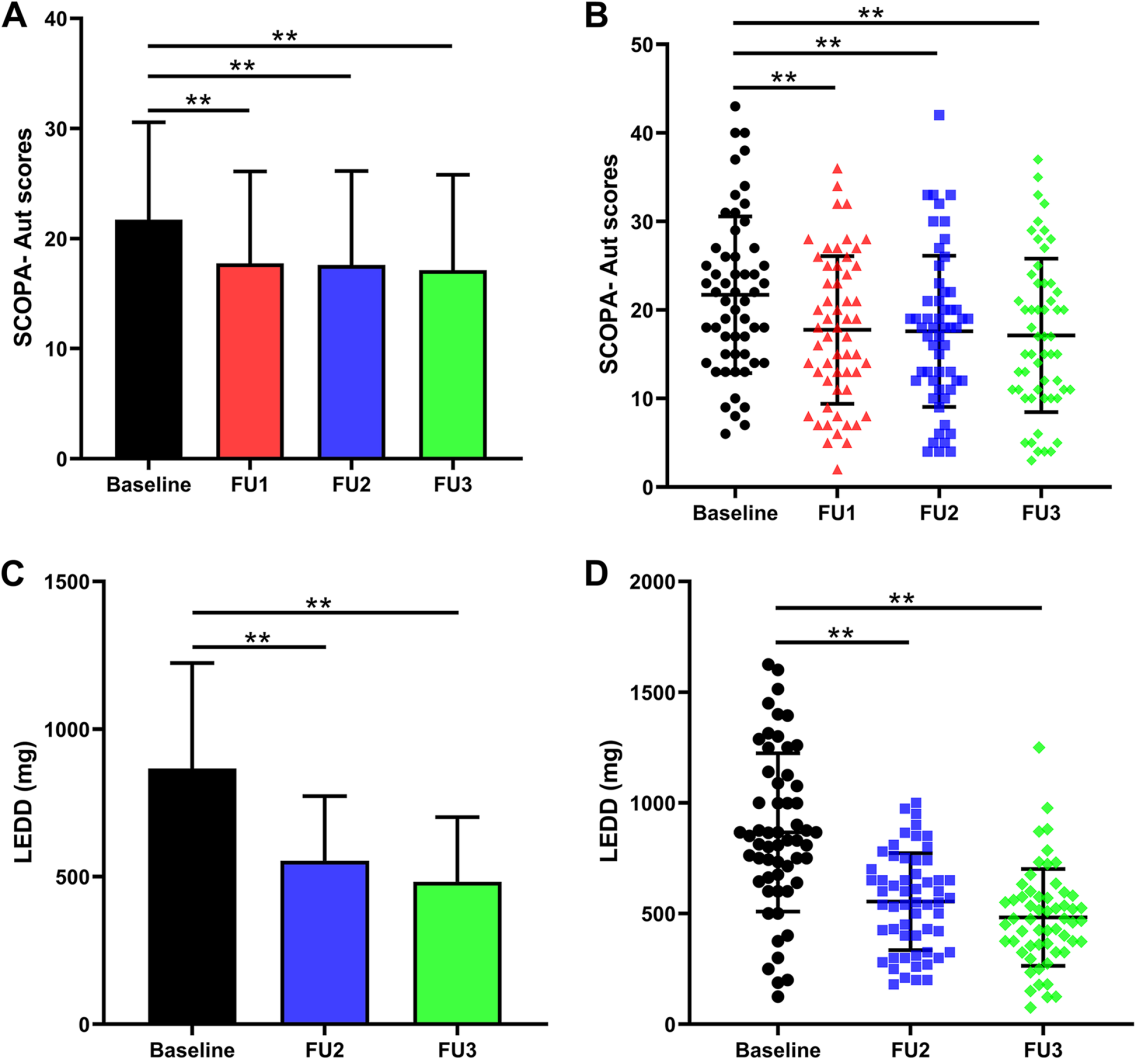
SCOPA- Aut and LEDD comparison between pre-and postoperative clinical state. **A**-**B** SCOPA- Aut scores were improved by 22.16%% 6 months after surgery; **C**-**D** the reduction rate of LEDD was 40.08% at 6 months after surgery(**: *P* < 0.001). (STN-DBS: subthalamic nucleus—deep brain stimulation; SCOPA-Aut: the Scale for Outcomes in PD for Autonomic Symptoms; LEDD: Levodopa equivalent dose) [Baseline: baseline; FU1: 1 month after surgery; FU2: 3 months after surgery; FU3: 6 months after surgery]

In this study, 57 patients were included, operated, and examined preoperatively, with planned follow-ups after 1, 3, and 6 months of continuous STN-DBS. Comparisons between preoperative and postoperative (1, 3, and 6 months after surgery) clinical stages are summarized in Table 1 and Fig. 1. At 6 months of follow-up, the SCOPA-Aut scores (scales for outcomes in PD autonomic symptoms) [M (IQR)] improved significantly [14.59% (18.32%), 24.00% (27.05%), 22.16% (27.07%), respectively, all *P* < 0.001] at 1, 3, and 6 months of STN-DBS, respectively. Analysis of the SCOPA-Aut sub-items showed significant improvements only in the urine [36.93% (60.00%)] and thermoregulation [40.00% (66.67%)] sub-items at 6 months after surgery (*P* < 0.001).

**Table 1:** Comparison of preoperative and postoperative clinical state [M (IQR)]

Time	Preoperative	Postoperative			Total *P*	*χ2*	*P*_*1*_	*P*_*2*_	*P*_*3*_
		**1 month**	**3 month**	**6 month**					
**SCOPA-Aut**									
SCOPA-Aut (0–69) total	22 (12)	18 (14)	18 (10)	16 (12)	0.003	5.399	0.001	0.001	< 0.001
Gastrointestinal (Q1–7)	4 (2.8)	4 (4)	4 (2.8)	4 (2)	0.107	2.133	0.062	0.193	0.065
Urinary (Q8–13)	7 (4)	4.5 (3.8)	4 (5)	4 (5)	** < 0.001**	11.062	** < 0.001**	** < 0.001**	** < 0.001**
Cardiovascular (Q14–16)	2 (1.8)	2 (1)	2 (1.7)	2 (2)	0.248	1.418	0.049	0.107	0.345
Thermoregulatory (Q17–21)	4 (2)	3 (2.8)	3 (3)	3 (2.8)	** < 0.001**	8.049	** < 0.001**	** < 0.001**	** < 0.001**
Pupillomotor (Q19)	1 (2)	1 (2)	1 (2)	1 (2)	0.264	1.362	0.159	0.073	0.088
Sexual (Q22–25)	2 (1.7)	2 (2)	2 (1)	2 (1.7)	0.792	0.346	0.659	0.871	0.542
**HAMA **(0–56)	16 (14)	11 (11)	9 (10)	11 (11)	** < 0.001**	12.839	** < 0.001**	** < 0.001**	** < 0.001**
**HAMD **(0–68)	14 (13)	9 (9)	9 (8)	9 (9)	** < 0.001**	11.664	**0.004**	** < 0.001**	** < 0.001**

P_1_, P_2_ and P_3_ values are, respectively, the results of comparisons between preoperative results and results 1, 3 and 6 months after surgery, *SCOPA-Aut* the Scale for Outcomes in PD for Autonomic Symptoms, *HAMA* Hamilton Anxiety Rating Scale, *HAMD* Hamilton Depression Rating Scale

As previously reported [5], UPDRS-III scores (medication-OFF) improved (55.42%), the PDQ-39 scores improved (47.39%), and the LEDD decreased by 40.08% at 6 months after surgery. The improvements of HAMA scores and HAMD scores of 57 patients were [41.7(34.9)% and 37.5(33.4)%, respectively (both, *P* < 0.001)].

### The correlation analyses

1. There were correlations between the improvements of SCOPA-Aut scores, LEDD, and PDQ-39 scores, and the improvements of UPDRS-III scores (Med-OFF) (Table 2). There was no significant correlation between the improvement of SCOPA-Aut scores and the improvement of UPDRS-III scores (Med-OFF) (*P* > 0.05).
2. There was a correlation between the improvement of SCOPA-Aut scores and the improvement of PDQ-39 scores (Table 2). There was no significant correlation between the improvement of SCOPA-Aut scores and the improvement of PDQ-39 scores (*P* > 0.05). There was no significant correlation between the improvement of urinary and thermoregulatory scores, and the improvement of PDQ-39 scores (all, *P* > 0.05) (Table 3).
3. There was a correlation between the improvement of SCOPA-Aut scores and the improvements of HAMA, and HAMD scores (Table 2). The improvement of SCOPA-Aut scores was positively correlated with the improvement of HAMA scores (*r* = 0.325, *P* = 0.015). The improvement of SCOPA-Aut scores was positively correlated with the improvement of HAMD scores (*r* = 0.265, *P* = 0.049), indicating that the better the improvement of anxiety and depression, the better the improvement of autonomic dysfunctions.
4. There were correlations of SCOPA-Aut scores, PDQ-39 scores, and clinical variables (Table 4). For SCOPA-Aut scores, there was no correlation with disease duration and Hoehn-Yahr grade (*P* > 0.05), but there was a positive correlation with age (*r* = 0.428, *P* = 0.001), indicating that the older the PD patients, the more serious the autonomic dysfunction symptoms. For PDQ-39 scores, there were positive correlations with disease duration (*r* = 0.296, *P* = 0.025) and Hoehn-Yahr grade (*r* = 0.366, *P* = 0.005).

**Table 2:** The correlation between the improvement rate of SCOPA-Aut scores, and clinical parameters 6 months after surgery

	**improvement rate (%)**	**the improvement rate of SCOPA-Aut scores (%)**	***r***	***P***
UPDRS-III (Med-off)	55.42	22.16	0.086	0.527
PDQ-39	47.39	22.16	0.126	0.355
HAMA	34.27	22.16	0.352	**0.015**
HAMD	26.95	22.16	0.265	**0.049**

**Table 3:** The correlation between the improvement rate of Urinary; Thermoregulat scores and the improvement rate of PDQ-39 scores 6 months after surgery

	**improvement rate (%)**	**the improvement rate of PDQ-39 scores (%)**	***r***	***P***
Urinary	36.93	47.39	0.013	0.926
Thermoregulat	40.00	47.39	0.022	0.875

**Table 4:** The correlation between SCOPA-Aut scores, PDQ-39 scores and clinical variables 6 months after surgery

	**Age**		**Disease duration**		**Hoehn-Yahr grade**	
	***r***	***P***	***r***	***P***	***r***	***P***
SCOPA-Aut	0.428	**0.001**	0.221	0.101	0.086	0.528
PDQ-39	**0.006**	0.967	0.296	**0.025**	0.366	**0.005**

## Discussion

DBS is widely used in the clinical treatment of PD because it is minimally invasive, adjustable, and reversible. Most PD patients experience autonomic dysfunctions at different clinical stages, with an incidence of 14% − 80% [11, 12]. Autonomic dysfunctions of PD patients include gastrointestinal malfunction (constipation, dysphagia, or choking); urinary disturbance (increased nocturia, frequent urination, and endless urination); sexual dysfunction (impotence, vaginal dryness, etc.); thermoregulatory aberrance (sweat, intolerance, etc.); cardiovascular dysregulation (postural hypotension and dizziness), and pupillo-motor and tear abnormalities [13], which seriously affect the QOL of PD patients. In this retrospective study of 57 PD patients treated with STN-DBS, using correlation analysis, it was found that the older the PD patient, the more serious the autonomic dysfunction. It was speculated that the older age of onset of Lewy bodies was related to the deposition of parts related to autonomic dysfunctions.

The mechanism for the improvement of symptoms of autonomic dysfunctions may be that STN-DBS directly regulates the basal ganglia-thalamus-cortical circuit, thereby regulating the thalamus, lateral frontal lobe, and anterior cingulate gyrus (the center of the autonomic nervous system) to improve the symptoms of autonomic dysfunctions [14]. The effect of STN-DBS on gastrointestinal dysfunction involves reducing constipation, dysphagia, and salivation [15]. Studies have shown that the effects of STN-DBS on urinary disturbance involved reducing detrusor muscle tension, and increasing bladder capacity and reflexing volume [16]. The severity of bladder dysfunction seems to be associated with relative degeneration of the caudate nucleus, among other areas [17]. STN-DBS can improve the temperature perception of PD patients, and DBS can improve hyperhidrosis and heat intolerance [18], which may be related to stimulation of the tail of STN, the ventral thalamus, and the zona incerta (ZI). The effect of STN-DBS on sexual dysfunction may be related to stimulations of the medial preoptic nucleus, anterior hypothalamic nucleus, and nucleus accumbens, resulting in changes in their activities [19]. The effect of STN-DBS on cardiovascular dysfunction involves increasing heart rate, and the sensitivity of baroreceptors and peripheral vascular tone, which can improve postural hypotension in PD patients [20], and which may be related to stimulating the limbic of STN or the ZI [21]. Frontal cortex, cingulate cortex, insula, thalami, basal ganglia, and periaqueductal grey matter may be related to gastrointestinal functions [22]. STN-DBS can activate the nerve fibers projecting from hypothalamus and crossing the subthalamic nucleus, that might be affecting gastrointestinal functions. Previous studies have shown that STN-DBS could improve autonomic dysfunctions in PD patients [23–27]. Few previous reports have used SCOPA-Aut to assess the effects of STN-DBS on autonomic dysfunctions. Previous studies have used NMSQ to assess NMS [28], However, this scale is only for NMSS, and not specifically for autonomic dysfunctions. The improvement of dysautonomic fluctuations after chronic stimulation was remarkable, especially for some symptoms. Urine showed the greatest reduction in the number of symptoms reported by patients after surgery. Regarding autonomic symptoms, the significant improvement of Scopa-Aut total scores after 6 months of STN-DBS, with the SCOPA-Aut total score increased by 22.16% (*P* < 0.001) after 6 months of follow-up, indicating that STN-DBS improved autonomic dysfunctions of PD patients in the short term. However, the results were not equal for all categories of symptoms. Analyses of each SCOPA-Aut domain showed significant improvements at 6 months only for the urinary and thermoregulatory dysfunctions, which showed a remarkable decrease after chronic stimulation. A study of 24 patients reported similar results, with Scopa-Aut improving after 3 months of follow-up, but with subsequent deterioration [29]. Further follow-up studies are necessary to confirm these results. A prospective study with preoperative and postoperative urodynamics would provide more detailed information about the effects of STN-DBS on bladder function. We could not predict the preoperative impact on SCOPA-Aut, but the parallel improvement of PDQ-39 scores were identified as a significant co-variate. This might imply that the degree of improvement of autonomic dysfunction played an important role in improving the QOL. However, there was no significant correlation between the improvement of SCOPA-Aut scores and the improvement of PDQ-39 scores (*P* > 0.05). Furthermore, there was no significant correlation between the improvement of urinary and thermoregulatory scores, and the improvement of PDQ-39 scores (all, *P* > 0.05), which may have been due to many factors involving the STN-DBS effects on the QOL, in addition to motor symptoms associated with other important roles. The NMS impact was also very important, not just the autonomic dysfunctions, such as anxiety, depression, numbness, pain, sleep disorders, cognitive impairment, hallucinations, and other influences, which were significant, so we need to consider many factors, and judge them comprehensively.

In the present study, there were negative results of cardiovascular, gastrointestinal, and sexual functions of autonomic dysfunctions after STN-DBS. It may be because STN-DBS had no direct effect on autonomic cardiac innervation or muscle vasoconstrictor neurons [30]. Gastrointestinal function depends not only on central but also peripheral autonomic structures, which might not be systems modulated by STN-DBS [31]. DBS-STN has no direct effect on the PD patients’ sexual life. Emotional well-being may contribute to a different opinion of the sexual function according to the patient’s sex [32].

In previous reports, SCOPA-Aut scores increased by age and disease duration [33]. In our study, SCOPA-Aut total scores increased by age, indicating that the autonomic dysfunction gradually worsened with age.

Autonomic dysfunction is related to emotional disorders, and may involve a common mechanism [34]. The 5-HT neurotransmitter system in patients with PD may be one of the mechanisms related to autonomic dysfunctions and emotional disorders. In our study, the improvement of SCOPA-Aut scores was positively correlated with the improvement of HAMA scores (*r* = 0.325, *P* = 0.015). The improvement of SCOPA-Aut scores was positively correlated with the improvement of HAMD scores (*r* = 0.265, *P* = 0.049), indicating that with improvements of anxiety and depression symptoms in PD disease, autonomic dysfunction would also improve.

The QOL dysfunctions deserve more focus, both in the preoperative and postoperative evaluations of PD patients for STN-DBS. Future studies should consider including these factors among the main outcomes, especially in studies focusing on optimal electrode location and tissue activation in STN, closed-loop DBS, directional electrodes, functional brain imaging, and brain networks.

### Limitations

There were some limitations in this study. (1) The sample was small and it was a retrospective study. (2) SCOPA-Aut scores for preoperative evaluation of autonomic dysfunctions were subjective. (3) The postoperative follow-up time was 6 months, which was short. (4) A large number of statistical tests were conducted without performing corrections for multiple testing.

## Conclusion

STN-DBS improved autonomic dysfunctions of PD, urinary, and thermoregulatory aspects of autonomic dysfunctions, which were postoperatively improved in the short term. There was a close relationship between improved autonomic dysfunctions and improved anxiety and depression at 6 months. We should therefore direct more attention to autonomic dysfunctions in PD patients, involving detailed preoperative evaluations and postoperative follow-ups, to improve the QOL of patients.

## Data Availability

The datasets used and/or analyzed during the current study not publicly available due to privacy reasons of patients, but are available from the corresponding author on reasonable request.

## Abbreviations

PD: Parkinson’s disease
STN: Subthalamic nucleus
DBS: Deep brain stimulation
UPDRS-III: Unified Parkinson’s Disease Rating Scale-Part III
LEDD: Levodopa equivalent day dose
QOL: Quality of life
PDQ-39: 39-Item Parkinson’s Disease Questionnaire
NMS: Non-motor symptom
SCOPA-Aut: The Scales for Outcomes in Parkinson’s Disease-Autonomic questionnaire
HAMA: The Hamilton Anxiety Rating Scale
HAMD: The Hamilton Depression Rating Scale
LCT: Levodopa challenge test
Med-off: Medication-off
Med-on: Medication-on
IPG: Implantable pulse generator
